# Atypical forms of diabetes mellitus in Africans and other non-European ethnic populations in low- and middle-income countries: a systematic literature review

**DOI:** 10.7189/jogh.09.020401

**Published:** 2019-12

**Authors:** Charlotte Bavuma, Diomira Sahabandu, Sanctus Musafiri, Ina Danquah, Ruth McQuillan, Sarah Wild

**Affiliations:** 1 University of Rwanda, College of Medicine and Health Sciences, Kigali University Teaching Hospital, Rwanda; 2 Institute for Social Medicine, Epidemiology and Health Economics, Charité – Universitaetsmedizin Berlin, Germany; 3 Department of Molecular Epidemiology, German Institute of Human Nutrition, Potsdam-Rehbruecke, Germany; 4 Usher Institute of Population Health Sciences and Informatics, University of Edinburgh, Edinburgh, UK; * Joint senior authors

## Abstract

**Background:**

Atypical presentations of diabetes mellitus (DM) have been reported in non-European ethnic populations under various names. It is unclear whether those names are used for the same or different clinical phenotypes. Unclear terminology may lead to inappropriate treatment and an underestimation of the burden caused by atypical diabetes phenotypes overlapping with classic types of diabetes. This review aimed to describe the terms used for atypical forms of diabetes and to investigate whether the terms are used for similar or different phenotypes.

**Methods:**

PubMed and Scopus were searched for relevant publications in French or English available before 15 September 2015 using the terms: ”Atypical diabetes”, “Malnutrition Related Diabetes Mellitus (MRDM)”, “Fibro-calculus pancreatic diabetes (FCPD)”, Protein deficient Pancreatic Diabetes (PDPD)”, “African diabetes”, “Ketosis prone-type 2 diabetes”, “tropical diabetes”, “Flatbush diabetes”, “J-type diabetes”. Titles, abstracts screening and quality assessment were performed by two independent authors. Observational studies addressing atypical diabetes in humans aged 14 years and above were included. One author extracted data from selected articles.

**Results:**

22 articles among 350 identified articles were retained for data extraction. Two atypical diabetes phenotypes were identified, each of them with a variety of names but similar definitions. One phenotype occurred in very thin people less than 30 years of age, typically from poor socio-economic backgrounds and requires insulin for life. It differs from type 1 diabetes in the tolerance of high blood glucose without ketosis in the absence of exogenous insulin. The second phenotype resembles type1 diabetes as it presents with ketosis at onset but responds well, as type2 diabetes, to oral hypoglycemic drugs after initial stabilization with insulin. It occurs in individuals who are usually over 30 years of age, with normal or overweight and absence of auto antibodies mainly found in type 1 diabetes.

**Conclusion:**

The scarce existing literature used various terms for similar diabetes phenotypes. Agreement on nomenclature for the various forms of diabetes using the above reported characteristics are needed in populations where atypical forms of diabetes exist as well as better characterization of phenotypes and genotypes to inform evidence based treatment.

Diabetes mellitus is a highly heterogeneous disease, the classification of which has changed as knowledge about its clinical and pathophysiological features have evolved [1-5]. In 1979, the National Diabetes Data Group (NDDG) established a classification based mainly on diabetes treatment requirements [1]. This classification included insulin dependent diabetes mellitus (IDDM), non-insulin dependent diabetes mellitus (NIDDM), gestational diabetes and diabetes secondary to other diseases like pancreatic cancer and other endocrine diseases [1]. In 1985, the World Health Organization (WHO) Study Group on Diabetes adopted the NDDG classification and malnutrition related diabetes mellitus (MRDM) was officially recognized [2]. MRDM was ranked as a standalone clinical subgroup with 2 subtypes, protein deficiency pancreatic diabetes (PDPD) and fibrocalculous pancreatic diabetes (FCPD), defined as diabetes onset in young people (less than 30 years of age) in developing countries, with a history of under nutrition, wasting and presence of pancreatic calculi or fibrosis in the FCPD [2]. However MRDM was subsequently dropped from official classifications as a consequence of lack of evidence by the International Expert Committee which revised the NDDG and WHO Study Group’s diabetes classification by introducing new nomenclature and modifications based on the etiology of diabetes [3]. This modified classification includes type 1 diabetes mellitus mainly due to auto-immune destruction of beta cells in the pancreas; type 2 diabetes whose etiology is essentially insulin resistance, gestational diabetes and secondary diabetes. Nevertheless, there are many forms of atypical diabetes which do not fit into the definition of these classic recognized types of diabetes. The picture is further complicated by the fact that there is no standardized nomenclature in the literature to describe atypical diabetes.

Since the early 1990s, the term atypical diabetes mellitus has been used to refer to some rare types of diabetes mellitus such as Maturity Onset Diabetes of the Young (MODY) [6]. MODY is a well-established entity characterized by an autosomal-dominant transmission, onset at a young age (below 40 years old), a strong family history of diabetes, slow progression, mild or absence of clinical symptoms or signs of diabetes and response to oral anti-diabetic treatment [7]. This form of diabetes resembles classic type 2 diabetes but differs in having a young age of onset and differs from type 1 diabetes by its slow onset, silent clinical manifestation, response to oral therapy and absence of islet auto-antibodies [7]. Latent Autoimmune Diabetes of Adult (LADA), also referred to as type 1.5 diabetes, is another atypical form, which resembles type 1 diabetes but with a late age of onset [8]. LADA may be mistakenly diagnosed as type 2 diabetes based on its clinical manifestation; however it differs from type 2 diabetes because of the presence of auto antibodies such as glutamic acid decarboxylase antibodies (anti-GAD) which are absent in type 2 diabetes [8].

LADA and MODY are well characterized and have long been recognized globally as atypical forms of diabetes. There are, however, other forms of atypical diabetes which do not clearly fit within the existing classifications. These forms appear to be more prevalent in populations of African and Asian ancestry. One such rare and atypical presentation of diabetes mellitus known as ketosis prone type 2 diabetes, has been described in people of non-white ancestry in European and American countries [9-12]. However, the scarcity of data on this form of diabetes from low and middle income countries (LMIC) makes it difficult to assess the prevalence of these forms of diabetes and to plan appropriate health services.

Terms such as “atypical diabetes”, “Malnutrition Related Diabetes Mellitus (MRDM)”, “Fibro-calculus pancreatic diabetes (FCPD)”, Protein deficient Pancreatic Diabetes (PDPD)”, “African diabetes”, “Ketosis prone-type 2 diabetes”, “tropical diabetes”, “Flatbush diabetes”, “J-type diabetes” among others have been used to describe diabetes phenotypes which do not clearly fit the type1 or type 2 diabetes definitions and which are not yet well understood. These terms are not, however, clearly defined in the literature, and it is unclear whether the same term is used to describe different phenotypes in different contexts or conversely, whether different terms are used to describe similar phenotypes in different contexts. This lack of clarity is problematic because it may lead to inappropriate diabetes classification at diagnosis, difficulties in the application of diabetes treatment guidelines (based on classic types of diabetes) and the provision of appropriate educational materials globally and particularly in underserved settings.

The aim of this study, whose protocol has been published in PROSPERO [13] is to systematically review available evidence on different terms used for atypical diabetes in order to clarify if they have the same or different definitions and to assess whether distinct phenotype(s) can be identified.

## METHODS

### Search strategy

PubMed and Scopus databases were searched to retrieve relevant publications related to the review objectives. The following key words were used to search relevant articles: Flatbush diabetes, ketosis-prone diabetes, tropical diabetes, malnutrition-related diabetes, fibrocalculous pancreatic diabetes, chronic pancreatic diabetes, protein deficiency pancreatic diabetes and African diabetes. The search strategy was adjusted as appropriate for each database. The search strategy is detailed in the Annexes S1 and S2 in **Online Supplementary Document**. References and citations of included studies were screened by one author (CB) to identify relevant documents.

### Inclusion and exclusion criteria

Observational studies including prospective or retrospective cohort studies, case–control, cross-sectional studies and case series published before the date of download (15 September 2015) on human non-European ethnic participants aged 14 years and above were included. We included studies in English or French reporting on atypical diabetes mellitus expressed as used key words. Studies with unavailable abstract, genetic studies, case reports and studies focusing on classic types of diabetes such as type 1 and type 2 diabetes mellitus, gestational diabetes mellitus, secondary diabetes, LADA and MODY were excluded.

### Selection and critical appraisal of studies

Titles and abstracts of retrieved bibliographic records were screened by two different authors (CB and DS). Disagreements were resolved by discussion among the two and consensus. The full text of each potentially eligible study was retrieved through HINARI and the library of Edinburgh University. Full texts were critically appraised independently by two authors (CB and DS). The quality of individual studies was assessed using the Health Evidence Bulletins – Wales checklist for assessing the quality of observational studies [14]. This tool was chosen because it can be used to assess different types of observational studies. Articles were retained, for data extraction, based on relevance to the review’s objectives, representative sample size to answer the research question and clear objectives and outcomes.

### Data extraction and report synthesis

Data extraction was done by one author (CB) using a specially designed worksheet including terms used to describe atypical forms of diabetes, its definitions clinical and biochemical characteristic. Study characteristics including date of publication, country and study method (sample size, type of study, setting) were extracted from individual studies. Terms identified that used the same definition and similar clinical characteristics were grouped in one phenotype. Narrative report was done by CB and reviewed by SM, ID, RM and SW.

## RESULTS

### Overview of included articles

**Figure 1:**
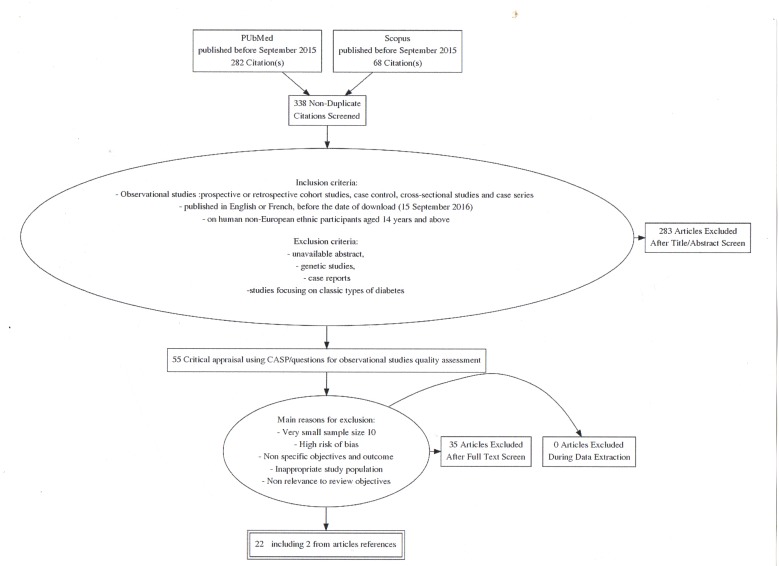
Flowchart showing inclusion process.

A total of 282 articles from PubMed and 68 from Scopus were identified. **Figure 1** describes the selection process. Following removal of duplicates there were 338 articles of which 55 were retained for quality assessment after titles and abstract screening against inclusion criteria and confirming relevance to the review objectives. Quality assessment led to the rejection of a further 35 articles and 20 were retained for data extraction and narrative synthesis with a further two studies identified from references. The main reasons for exclusion of full text at appraisal stage were high risk of bias, inappropriate study population in terms of sample size and participants’ selection, unclear objectives and outcomes and lack of definition of atypical diabetes terminology. The final number of articles remaining for analysis was 22.

Most of the articles included (12 out of the 22) were published before 2000 as shown in **Table 1**. The majority of studies were from various Asian countries (13 among 22 included records) mainly from India. There was a scarcity of data from African countries, Latin America and the Caribbean; there were three clinic-based studies with a small sample sizes from Ethiopia and three from Jamaica using different terms for atypical diabetes, with small sample size and different populations. One study from Ethiopia was included even though it did not have the definition of any form of atypical presentation but did contribute data on the clinical characteristics of a young sub-Saharan population with diabetes requiring insulin [30]. All studies included participants with diagnosed diabetes; however there was considerable heterogeneity in the age groups of study participants. Some studies included participants aged 35 years or less [18,19,23,24], while others included participants of all ages. In addition, the included studies had a variety of objectives and outcomes that made comparison difficult. Nevertheless, the included studies addressed various presentation of diabetes which might help to clarify the different terms used for atypical presentation of diabetes and to define a phenotype or phenotypes associated with these terms. **Table 1** provides a summary of the characteristics of the included articles.

**Table 1:** Characteristics of included studies sorted by date of publication

First author name (reference number)	Year of publication	Country	Study design	Sample size
Mohan [15]	1983	India	case-control study	control +23, NIDDM: 42, IDDM: 40, TPD: 42
Mohan [16]	1985	South India	case-control, clinic based	33 TPD; 35 type 2 (NIDDM); 35 non diabetic; all matched for age, sex, duration of diabetes; consecutive inclusion on 2 y
Mohan [17]	1985	South India	Case-control, hospital based	20 TPD, 20 IDDM, 20 MODY age and sex matched
Vannasaeng [18][	1986	Thailand	case-control, clinic based	13 CTPD, 23 IDDM, 18 NIDDM and 10 non-obese individual without diabetes
Ramachandran [19]	1987	South India	cross-sectional, hospital based	545 patients with diabetes (461 included, 80 unclassified and 4 GD excluded)
Abdulkadir [20]	1990	Ethiopia	cross-sectional and case-control, clinic based	63 MRDM, 18 type 1, 19 type 2, 6 MRDM were excluded in hormones work up because not fitting MRDM definition
Ragoobirsingh [21]	1990	Jamaica	case-control study	13 PIDDM, 11 IDDM, 10 NIDDM and 12 normal subject
Morrison [22]	1992	Jamaica	case-control clinic base study	14 PIDDM, 10 IDDM, 10 NIDDM, 10 normal control
Bhatia [23]	1995	North India	case-control, clinic based	20 consecutive FCPD, 19 PDPD, 20 Patients with TYPE 1 DM, 32 nealthy
Dabadghao [24]	1996	North India	case-control clinic base	23 PDPD; 25 FCPD; 62 type 1
Ragoobirsingh [25]	1997	Jamaica	case-control	14 PIDDM, 10 IDDM, 10 NIDDM and 10 healthy controls
Mohan[26]	1998	South India	case-control study	57 FCPD, 40 Type 1 DM, 71 Type 2 DM, 45 healthy non diabetic patients
Mauvais-Jarvis[12]	2004	France	cohort study	111 Sub-Saharan origin individuals with ketosis-prone type 2 diabetes, 21 with type 1 diabetes and 88 with type 2 diabetes
Maldonado [27]	2005	USA	cohort study, tertiary hospital based study	106 patients with ketosis-prone diabetes
Otiniano [28]	2005	USA	cross-sectional	172 patients set in 2 groups: 1 group with metabolic syndrome as par WHO definition and 1 group with metabolic syndrome
Balasubramanyam [29]	2006	USA	longitudinal case-control study(31month follow up)	294 patients with DKA, all ages and gender
Fekadu S[30]	2010	Ethiopia	case-control clinic base(Multi center clinic based)	107 patients 110 controls
Liu [31]	2013	Chine	case-control clinic based study	159 overall patients from which 79 with ketosis onset diabetes and 80 KPD
Seok [32]	2013	Korea	3 tertiary centers based Cohort study (4years follow up)	60 newly diagnosed KPD
Gupta [33]	2014	Thailand	case-control	20KPD, 12 type 1 DM
Yotsapon [34]	2014	Thailand	cohort study, 24months follow up clinic based	20 KPD and 12 type 1 DM
Zhang [35]	2015	China	cross-sectional study	238 individuals with diabetes from inpatients department

NIDDM – non-insulin dependent diabetes mellitus, IDDM – insulin dependent diabetes mellitus, TPD – tropical pancreatic diabetes, MODY – maturity onset diabetes of the young, CTPD – calcific tropical pancreatic diabetes, GD – gestational diabetes, MRDM – malnutrition-related diabetes mellitus, FCPD – fibro-calculous pancreatic diabetes, PDPD – protein deficient pancreatic diabetes, DKA – diabetic ketoacidosis, KPD – ketosis-prone diabetes, PIDDM – phasic insulin dependent diabetes mellitus

### Names and definitions of atypical forms of diabetes from the literature

In 1983, Mohan and collaborators used the term “tropical pancreatic diabetes (TPD)” for diabetes mellitus occurring in individuals aged 15 to 30 years who were underweight or wasted and required insulin but who did not experience ketosis on discontinuation of insulin treatment; this phenotype is also referred to as ketosis resistant diabetes [15]. In subsequent years, the same author and other scientists added new criteria to the definition of TPD: history of chronic abdominal pain from childhood, absence of potential causes of pancreatic calcification such as alcohol consumption, gall stones and other biliary obstructive diseases or high parathyroid hormones; and presence of pancreatic calcification on plain abdominal radiography [16,17]. Subsequently, other authors have used different terms for a similar phenotype: calcific tropical pancreatic diabetes (CTPD) with addition of symptoms or signs of under nutrition [18], fibro-calculus pancreatic diabetes (FCPD) with an addition of fibrosis and biliary duct dilatation identified on abdominal ultrasound [19,23,24,26].

The above phenotype under various terminologies was described in some studies as a subclass of malnutrition related diabetes (MRDM), the term recommended by WHO in 1985 [19,23,24]. However, in one report, MRDM was used with a global definition including diabetes in individuals with poor socioeconomic status associated with one or more of the following: at least 3 months duration of typical symptoms of diabetes, leanness with underweight (BMI≤18.5 kg/m^2^) at diabetes onset, insulin requirement from diagnosis for blood glucose control and absence of significant ketonuria [20]. MRDM has been reported to also cover protein deficient pancreatic diabetes (PDPD) with a similar definition [19]; and with the addition of diabetes onset below the age of 30 years, lower age of onset of “TPD” and severe hyperglycemia usually greater than 11.2 mmol/dL in other studies [23,24]. Different names such as “J type diabetes”, phasic insulin dependent diabetes mellitus (PIDDM) and ketosis resistant diabetes have been used in different studies for phenotypes similar to PDPD with an additional criterion of high daily insulin requirement which may denote insulin resistance [16,21,22,25].

Ketosis prone diabetes (KPD) has been defined as a subgroup of TPD with the addition of increased susceptibility to developing ketosis [16]. However KPD has been used in other studies of residents and emigrants from LMIC to denote newly diagnosed diabetes, usually after the age of 30 years of age, with symptoms and signs of diabetes, unprovoked ketosis (absence of provoking factor for ketosis such as infection), transient insulin requirement and absence of islet cells (ICAs Ab) and glutamic acid decarboxylase anti-bodies (GADAb) [12,29,32,34,35]. This phenotype is variously described as ketosis prone diabetes [28,31,33] or ketosis prone type 2 diabetes [12]. These names have been used interchangeably in the same article by some authors [27]. Flatbush diabetes, atypical diabetes and type 1.5 diabetes were also reported as synonyms for ketosis prone diabetes or ketosis prone type 2 diabetes [35]. Furthermore, subgroups of patients presenting with ketosis have been defined: ketosis prone diabetes type 1a which is defined as equivalent to classic diabetes type 1a characterized by autoimmune destruction of beta cells in the pancreas, ketosis prone type 1b defined as diabetes with non autoimmune beta cells function failure, ketosis prone type 2a and ketosis prone type 2b respectively characterized by preserved beta cell function and with presence of autoantibodies in the type 2a and without antibodies in type 2b [29]. In another study, subgroups of KPD were defined using different names, such as KPDM-insulin in which diabetes could be controlled after discontinuing insulin and using alternative treatments and KPDM+insulin characterized by insulin requirement for life in order to manage hyperglycaemia [33].

### Phenotypes and clinical profile

From the above definitions numerous names were used for similar phenotypes which could collectively be described as either malnutrition related diabetes (MRDM) or ketosis prone diabetes whose characteristics are summarized in **Table 2**.

**Table 2:** Identified phenotypes’ characteristics and classic types of diabetes

Characteristic	MRDM		Type1 DM	Ketosis-prone type 2 DM	Type 2 DM
Subtypes	PDPD	FCPD			
Onset age	Third decade or early adulthood (≤30 y)	Fourth decade or early adulthood (≤40 y)	Mostly less than 18 y	Third and fourth decade	Majority in fourth decade
Family history of diabetes	Weak⃰	Weak⃰	Moderate†	Strong‡	Strong‡
History of childhood malnutrition	Strong§	Strong§	-	-	-
Body mass index (5kg/m^2^)	Low (<18.5)	Low (<18.5)	Normal (18.5-24.9)	Overweight (25-29.9) or obese (≥30)	Overweight (25-29.9) or obese (≥30)
Hyperglycemia at diagnosis	Severe	Severe	Moderate	Severe	Moderate
Chronic abdominal pain	No	Yes	No	No	No
Ketosis in urine in absence of insulin treatment	Absent	Absent	Present, often with triggering factor	Present, without triggering factors	Absent
Treatment requirement	Insulin dependent	Insulin dependent	Insulin dependent	Requiring insulin at onset and responding to oral therapy after one to two years	Non-insulin dependent
Calcification, bile duct dilation or decreased size of pancreas on imaging	Absent	Present	Absent	Absent	Absent
Beta cell function	Impaired	Impaired	Impaired	Reserved with improvement after stabilization of Glucose level at onset	Reserved with impairment with progression of disease
Exocrine pancreatic deficiency	Rare	Frequent	Absent	Absent	Absent

PDPD – protein deficient pancreatic diabetes, MRDM – malnutrition-related diabetes mellitus, DM – diabetes mellitus, FCPD – fibro-calculous pancreatic diabetes, y – years

*No association with family history of diabetes, sporadic case.

†Moderate association with family history of diabetes, often first degree family members are affected [36].

‡Strong association with family history of diabetes.

§Strong association with a history of childhood malnutrition.

MRDM has been described under the following names: “tropical pancreatic diabetes”, “chronic tropical pancreatic diabetes”, “fibro-calculus pancreatic diabetes”, “protein deficient pancreatic diabetes”, “J type diabetes” and “phasic insulin dependent diabetes (PIDDM)”. Most of these publications were before 2000, apart from some papers from Ethiopia describing atypical presentation of insulin requiring diabetes [30]. The common characteristics of this phenotype are the occurrence of diabetes in abnormally lean young people from poor socioeconomic conditions; with “type 1 like” diabetes at presentation but without ketoacidosis and the potential for some people to manage hyperglycaemia after the acute phase in which insulin requirements are high with non-insulin treatments.

The only study identified describing the prevalence of MRDM estimated that it occurred in 6% of Indian diabetic patients ≤30 years old [19]. The age of onset was typically found to be within the third decade of life, which is the between median age of onset of type 1 diabetes and that of type 2 DM [19,20,23,24,26]. For example in one study, the mean (±standard deviation) age at onset was 23.6 ± 4.4 years in this phenotype (MRDM) while it was 14.5 ± 7.6 in type 1 DM [24]. As most of the studies included participants aged 30 years or less and thereby excluded those who had their diabetes diagnosed after that age, the prevalence of MRDM may be underestimated. Fekadu and collaborators in Ethiopia found a male preponderance and poor socioeconomic conditions in their insulin requiring patients in their study of the atypical diabetes phenotypes [30]. The atypical presentation of diabetes, in abnormally thin people, appeared to be also characterized by lower body mass index at onset of diabetes in comparison to other classic types of diabetes [18,19,26], features of chronic malnutrition such as disproportionate skeletal growth, parotid enlargement, skin changes and/or scalp hair changes [20,30]. However Ramachandran and co-authors found low BMI in both people with type 1 and with atypical diabetes without other features of malnutrition related diabetes in their study, which was conducted in an urban tertiary level health facility in India. Presumably, this difference might relate to differences between populations in prevalence of malnutrition and/or differences in research settings (tertiary vs primary health facilities). Other characteristics reported by one or two studies, were the requirement of high daily insulin dose to control blood glucose [23] and lack of family history of diabetes [19].

MRDM, as described in 1985 by the WHO, had two sub-types: protein deficient pancreatic diabetes (PDPD) and fibrocalculus pancreatic diabetes, with the latter characterized by impairment of exocrine pancreas and changes on pancreas imaging such as calcification in the pancreas or fibrosis, biliary duct dilatation and decreased size and irregular surface of the pancreas [17,22,23]. Very few studies with small sample sizes evaluated the secretion of insulin by beta cell function in this MRDM phenotype and reported a better beta cell function than in classic type 1 but lower than in type 2 DM [15,16,18,19,23]. However a report from Ethiopia recorded similar beta cell function in this phenotype and type1 diabetes with lower beta cell function than in type 2 diabetes. Antibodies were reported to be rare in this phenotype in one clinic-based study without a comparator group [24].

Key features of MRDM phenotype described above are in line with the1985 WHO definition: onset below the age of 30 years, living in poverty, lack of ketosis in presence of very high blood glucose, low BMI, relative presence of features of under nutrition, requirement of high dose of insulin to control blood glucose, relative beta cell impairment with fibrocalcific pancreatic changes in a subset of patients.

In contrast, ketosis prone diabetes differs from MRDM in age at onset, higher BMI and presence of ketosis at onset. The age at diagnosis of KPD is reported to be later (in 4th decade) compared to that in type 1 DM [9,20,26] whose onset is typically around first and second decade. Family history of diabetes and male preponderance have been found to be frequent in this phenotype as well as higher body mass index than that in type 1 but similar to that in classic type 2 Diabetes [12,29,35]. The onset of this phenotype is accompanied by ketosis without provoking factors and insulin discontinuation for other treatment options across the time of follow up (3 to 12 months from onset) [32]. Permanent insulin treatment was recorded in a set of patients with this phenotype and those patients were characterized by impaired beta cell function [12]. Patients with older age at onset, higher level of endogenous insulin and metabolic syndrome were more likely to be able to switch from insulin to other treatments for diabetes [27,28], in two studies on multiethnic groups in USA without comparators from elsewhere. Furthermore, patients with KPD are described as having beta cell function reserve between that of classic type 1 and type 2 as expressed by both fasting and stimulated C peptide levels [31-35].

## DISCUSSION

This systematic review identified heterogeneous studies describing characteristics of people with atypical diabetes. Two main phenotypes have been identified that have been described using a variety of names. The first phenotype that we have identified as MRDM has some similar characteristics to the type 1 diabetes. It occurs in lean people living in poor socioeconomic conditions whose diagnosis is made at an older age (third decade) than most people with type 1 diabetes, who have features of under nutrition, absence of beta cell antibodies, who may have pancreatic calcification on imaging and who do not develop ketosis. Characteristics of MRDM have been reported mainly by a few historical clinic based studies with small sample sizes from LMIC with noticeable scarcity of data from Africa. The second phenotype, KPT2DM typically occurs in normal weight or overweight people who present with unprovoked ketosis at onset but in whom insulin may be withdrawn and replaced with other diabetes treatments after some months of glucose stabilization and has been reported to occur in Asian countries and in people of African ancestry in developed countries.

There is a scarcity of contemporary data on prevalence of MRDM. The existing literature from clinics based research in urban tertiary settings suggests that it is rare [19]. For example no cases of this phenotype were observed in 550 patients seen at Yaoundé central hospital in Cameroon [37]. However prevalence would be expected to be higher in underserved rural populations. Most qualified health professionals who might generate research questions and lead research projects are located in urban settings and have high clinical workloads, with limited opportunity and funding for conducting research. In addition, diabetes and non-communicable diseases in general suffer from persistent inequities in global health research funding, which continues to favor infectious diseases. Against this background, it is conceivable that cases of MRDM in rural clinics might be misclassified as classic type 1 diabetes. The implications for patients are significant, if such misclassification results in inappropriate treatment based on guidelines for type 1 diabetes.

In order to ensure that appropriate treatment guidelines are being followed and to build responsive and appropriate health systems, it is important to understand the contribution of different types of diabetes to a nation‘s overall diabetes burden. The situation is particularly challenging for health system planners in rapidly developing low- and middle-income countries (LMIC), who are already grappling with an increasing prevalence of type 2 diabetes, driven by a substantial increase in the prevalence of obesity and sedentary lifestyles.

MRDM typically occurs in the 3rd decade of life, which is older than the age at which type 1 diabetes typically occurs. Childhood under nutrition might be one of the reasons why diabetes mellitus is increasing in the young population in LMIC. For example Shen and collaborators reported that pre-diabetes and diabetes were common in young, lean South Asians in a multi-center population based study [38]. Furthermore, a case-control study from Jamaica showed that beta cell impairment and insulin sensitivity in adults with a history of under-nutrition varied by type of malnutrition and was worse in adults with a history of marasmus than those with a history of kwashiorkor [39].

Low body mass index (BMI) at onset was recorded to be frequent in MRDM and considered as a criterion for MRDM diagnosis by some studies. However one study recorded that body mass index was similar in people diagnosed with MRDM and classic type 1 DM. This discrepancy might be due to different study populations at different stage of disease at diagnosis and underlying BMI distributions in populations. However low BMI at onset in such poor patients may be due to many factors such as long duration of diabetes symptoms, as MRDM patients have been recorded to tolerate high blood glucose, exocrine pancreatic failure with nutrient malabsorption leading to wasting or persistent malnutrition from childhood sustained by hunger and poverty. Although there is a relationship between low body mass index and MRDM, there is a need to clarify whether MRDM contributes to weight loss.

One study addressing MRDM highlighted the low binding of insulin to blood cells, which reflects insulin resistance and that is consistent with longitudinal evidence which supports the correlation between low birth weight and cardiovascular risks including insulin resistance known to cause diabetes [40]. The pathophysiological mechanism of insulin resistance might be the same and may explain the high insulin daily dose required for blood glucose control in this phenotype but more research to confirm this hypothesis is needed. Patients with this phenotype may be managed as classic type 1 with additional consideration of their nutritional needs and exocrine pancreatic failure but more research is required to identify optimal treatments and to define the appropriateness of changing treatment.

To the best of our knowledge, only one study has described complications of the MRDM phenotype: a case-control study with a small sample, reported higher mean level of albuminuria in that phenotype than in IDDM (Type 1) and NIDDM (type2) (PIDDM: 153.1 ± 48.3; 37.7 ± 15.8 in NIDDM and 38.6 ± 15.8 in IDDM; *P* < 0.05) as well as decreased insulin binding to red and white blood cells together with lack of insulin in the atypical phenotype [22].

The findings are consistent with the WHO population based stepwise non-communicable disease risk factors survey in Rwanda which found a high prevalence of albuminuria in rural areas (with frequent childhood under nutrition) compared to semi-urban and urban area [41]. This suggests that patients with this phenotype would be at particularly high risk of developing diabetic nephropathy. However there is a lack of evidence on complications of diabetes in this population and the effectiveness of interventions.

Ketosis-prone type 2 diabetes includes features of both type 1 and type 2 diabetes. It resembles type 1 DM by the presence of ketosis at diagnosis; and type 2 DM in terms of later age and association with overweight at onset as well as preserved insulin secretion. Ketosis-prone type 2 diabetes has been described in African American populations and African immigrants to Europe. However cases have also been reported in European ancestry populations [42]. Very few studies from Africa have described this phenotype, although around 30% of patients presenting with hyperglycaemic crisis in an urban tertiary hospital in Cameroon were identified as having KPD [43]. In terms of the presence of antibodies and pancreatic beta cell function, ketosis prone type2 DM has been attributed the category of A-β+ (antibody negative and preserved beta cell function). This makes it different from LADA which is described as A+ β+. Despite many publications describing this phenotype, there is no consensus yet about whether it should be considered in current classification systems and under which name. Furthermore, existing treatment guidelines do not address its management and the risk of complications is not clear.

## CONCLUSION

Diabetes classification has evolved continuously however its presentation is heterogeneous in LMICs where there is limited information available about less common forms of diabetes. The lack of consensus on the naming and classification of diabetes occurring in abnormally thin young people in poor socioeconomic areas may overestimate type 1 DM prevalence and may result in inappropriate management of the subset of individuals with atypical diabetes. Further translational research to characterize diabetes, using reported characteristics in populations living in poverty with prevalent childhood malnutrition, is required.

Ketosis-prone diabetes phenotype, “type 2 DM-like with unprovoked ketosis at onset”, appears to exist in association with modernization and urbanization. It is unclear whether this can be considered as a subtype of type 2 DM and managed accordingly. A systematic review of evidence on the pathophysiology of this phenotype and further studies on adequate sample size to identify potential reasons to ketosis at diabetes onset and the duration of insulin requirement are required to provide evidence for its management.

## Additional material

Online Supplementary Document
