# Supplementary material for: Atypical forms of diabetes mellitus in Africans and other non-European ethnic populations in low- and middle-income countries: a systematic literature review
Source: J Glob Health. 2019 Jul 21;9(2):020401. doi: 10.7189/jogh.09.020401 (PMC6818125; doi:10.7189/jogh.09.020401)
Supplement: Online Supplementary Document [file jogh-09-020401-s001.pdf]

J Glob Health 2019; 9: 020401

Online Supplement S1: Search strategy

Search	Search Query	Items found
#11	Search (((((((("protein deficient pancreatic diabetes") OR "malnutrition related diabetes") OR "chronic calculus pancreatic diabetes") OR "fibrocalculus pancreatic diabetes") OR "tropical pancreatic diabetes") OR "african diabetes") OR "ketosis prone type 2 diabetes") OR "ketosis prone diabetes") OR "flatbush diabetes") OR "tropical diabetes"	282
#10	Search "protein deficient pancreatic diabetes"	10
#9	Search "malnutrition related diabetes"	70
#8	Search "chronic calculus pancreatic diabetes"	74
#7	Search "fibrocalculus pancreatic diabetes"	5
#6	Search "tropical pancreatic diabetes"	20
#5	Search "african diabetes"	10
#4	Search "ketosis prone type 2 diabetes"	26
#3	Search "ketosis prone diabetes"	70
#2	Search "flatbush diabetes"	7
#1	Search "tropical diabetes"	19

Supplement S2 : Scopus Search results

1. Murunga, A.N., Owira, P.M.O.
Diabetic ketoacidosis: An overlooked child killer in sub-Saharan Africa?
(2013) Tropical Medicine and International Health, 18 (11), pp. 1357-1364.

Document Type: Review
Source: Scopus

----

2. Ntsekhe, M., Damasceno, A.
Recent advances in the epidemiology, outcome, and prevention of myocardial infarction and stroke in sub-Saharan Africa
(2013) Heart, 99 (17), pp. 1230-1235. Cited 4 times.

Document Type: Article
Source: Scopus

----

3. Frank, L.K., Heraclides, A., Danquah, I., Bedu-Addo, G., Mockenhaupt, F.P., Schulze, M.B.
Measures of general and central obesity and risk of type 2 diabetes in a Ghanaian population
(2013) Tropical Medicine and International Health, 18 (2), pp. 141-151. Cited 1 time.

Document Type: Article
Source: Scopus

----

4. Lozano, R., Naghavi, M., Foreman, K., Lim, S., Shibuya, K., Global and regional mortality from 235 causes of death for 20 age groups in 1990 and 2010: A systematic analysis for the Global Burden of Disease Study 2010
(2012) The Lancet, 380 (9859), pp. 2095-2128. Cited 278 times.

Document Type: Article
Source: Scopus

----

5. Crowther, N.J., Norris, S.A.
The Current Waist Circumference Cut Point Used for the Diagnosis of Metabolic Syndrome in Sub-Saharan African Women Is Not Appropriate
(2012) PLoS ONE, 7 (11), art. no. e48883, . Cited 2 times.

Document Type: Article
Source: Scopus

----

6. Damasceno, A., Mayosi, B.M., Sani, M., Ogah, O.S., Mondo, C., Ojji, D., Dzudie, A., Kouam, C., Suliman, A., Schrueder, N., Yonga, G., Ba, S.A., Maru, F., Alemayehu, B., Edwards, C., Davison, B.A., Cotter, G., Sliwa, K.
The causes, treatment, and outcome of acute heart failure in 1006 Africans from 9 countries: Results of the sub-Saharan Africa survey of heart failure
(2012) Archives of Internal Medicine, 172 (18), pp. 1386-1394. Cited 22 times.

Document Type: Article
Source: Scopus

7.Crowther, N.J.
Early determinants of chronic disease in developing countries
(2012) Best Practice and Research: Clinical Endocrinology and Metabolism, 26 (5), pp. 655-665. Cited 1 time.

Document Type: Review
Source: Scopus

8.Mekong, J.O., Kengne, A.P., Dehayem, M.Y., Sobngwi, E., Mbanya, J.C.
Cardiovascular preventative therapies and outcomes of care among urban sub-saharan Africans with

type 2 diabetes: A cross-sectional study in Cameroon
(2012) Journal of Clinical Outcomes Management, 19 (10), pp. 446-452. Cited 1 time.

Document Type: Article
Source: Scopus

----

9. Ama, V., Kengne, A.P., Nansseu, N.J.R., Nouthe, B., Sobngwi, E.
Would sickle cell trait influence the metabolic control in sub-Saharan individuals with type 2 diabetes?
(2012) Diabetic Medicine, 29 (9), pp. e334-e337. Cited 1 time.

Document Type: Article
Source: Scopus

----

10. Shavadia, J., Yonga, G., Otieno, H.
A prospective review of acute coronary syndromes in an urban hospital in sub-Saharan Africa
(2012) Cardiovascular Journal of Africa, 23 (6), pp. 318-321. Cited 1 time.

Document Type: Review
Source: Scopus

----

11. Brun, R., Blum, J.
Human African Trypanosomiasis
(2012) Infectious Disease Clinics of North America, 26 (2), pp. 261-273. Cited 5 times.

Document Type: Review
Source: Scopus

12.Konin, C., Adoh, M., Adoubi, A., Ekou, A., Koffi, J., N'Djessan, J.J., Ahoua, A.
Pulse pressure monitoring in hypertensive black Africans [Intérêt du suivi de la pression pulsée chez l'hypertendu noir Africain]
(2012) Cardiovascular Journal of Africa, 23 (1), pp. e1-e6. Cited 1 time.

Document Type: Article
Source: Scopus

----

13. Ameh, J., Godwin, I., Obi, I., Puepet, F., Aminu, B., Suleiman, T.
The search for mitochondrial tRNA Leu(UUR) A3243G mutation among type 2 diabetes mellitus patients in the Nigerian population
(2011) African Journal of Biotechnology, 10 (62), pp. 13383-13389.

Document Type: Article
Source: Scopus

14.Urea-Torres, P., Metzger, M., Haymann, J.P., Karras, A., Boffa, J.-J., Flamant, M., Vrtovsnik, F., Gauci, C., Froissart, M., Houillier, P., Stengel, B.
Association of kidney function, vitamin D deficiency, and circulating markers of mineral and bone disorders in CKD
(2011) American Journal of Kidney Diseases, 58 (4), pp. 544-553. Cited 22 times.

Document Type: Article
Source: Scopus

15.Kolapo, K.O., Vento, S.
Stroke: A realistic approach to a growing problem in sub-Saharan Africa is urgently needed
(2011) Tropical Medicine and International Health, 16 (6), pp. 707-710. Cited 2 times.

Document Type: Article
Source: Scopus

16.Klimentidis, Y.C., Abrams, M., Wang, J., Fernandez, J.R., Allison, D.B.
Natural selection at genomic regions associated with obesity and type-2 diabetes: East Asians and sub-Saharan Africans exhibit high levels of differentiation at type-2 diabetes regions
(2011) Human Genetics, 129 (4), pp. 407-418. Cited 17 times.

Document Type: Article
Source: Scopus

----

14. Tuei, V.C., Maiyoh, G.K., Ha, C.-E.
Type 2 diabetes mellitus and obesity in sub-Saharan Africa
(2010) Diabetes/Metabolism Research and Reviews, 26 (6), pp. 433-445. Cited 12 times.

Document Type: Review
Source: Scopus

----

15. Mbanya, J.C.N., Motala, A.A., Sobngwi, E., Assah, F.K., Enoru, S.T.
Diabetes in sub-Saharan Africa
(2010) The Lancet, 375 (9733), pp. 2254-2266. Cited 82 times.

Document Type: Review
Source: Scopus

----

16. Agyemang, C., Addo, J., Bhopal, R., de Graft Aikins, A., Stronks, K.
Cardiovascular disease, diabetes and established risk factors among populations of sub-Saharan African descent in Europe: A literature review
(2009) Globalization and Health, 5, art. no. 7, . Cited 42 times.

Document Type: Review
Source: Scopus

----

17. Bischoff, A., Ekoe, T., Perone, N., Slama, S., Loutan, L.
Chronic disease management in Sub-Saharan Africa: Whose business is it?
(2009) International Journal of Environmental Research and Public Health, 6 (8), pp. 2258-2270. Cited 11 times.

Document Type: Review
Source: Scopus

----

18. Ntusi, N.B.A., Mayosi, B.M.
Epidemiology of heart failure in sub-Saharan Africa
(2009) Expert Review of Cardiovascular Therapy, 7 (2), pp. 169-180. Cited 32 times.

Document Type: Review
Source: Scopus

----

19. Majaliwa, E.S., Elusiyan, B.E.J., Adesiyun, O.O., Laigong, P., Adeniran, A.K., Kandi, C.M., Yarhere, I., Limbe, S.M., Iughetti, L.
Type 1 diabetes mellitus in the African population: Epidemiology and management challenges
(2008) Acta Biomedica de l'Ateneo Parmense, 79 (3), pp. 255-259. Cited 13 times.

Document Type: Article
Source: Scopus

----

20. Levitt, N.S.
Diabetes in Africa: Epidemiology, management and healthcare challenges
(2008) Heart, 94 (11), pp. 1376-1382. Cited 63 times.

Document Type: Review
Source: Scopus

----

21. Duvivier, C., Ghosn, J., Assoumou, L., Soulié, C., Peytavin, G., Calvez, V., Génin, M.A., Molina, J.-M., Bouchaud, O., Katlama, C., Costagliola, D.
Initial therapy with nucleoside reverse transcriptase inhibitor-containing regimens is more effective than with regimens that spare them with no difference in short-term fat distribution: Hippocampe-ANRS 121 Trial
(2008) Journal of Antimicrobial Chemotherapy, 62 (4), pp. 797-808. Cited 12 times.

Document Type: Article
Source: Scopus

----

22. Sobngwi, E., Choukem, S.P., Agbalika, F., Blondeau, B., Fetita, L.-S., Lebbe, C., Thiam, D., Cattan, P., Larghero, J., Foufelle, F., Ferre, P., Vexiau, P., Calvo, F., Gautier, J.-F.
Ketosis-prone type 2 diabetes mellitus and human herpesvirus 8 infection in sub-Saharan Africans
(2008) JAMA - Journal of the American Medical Association, 299 (23), pp. 2770-2776. Cited 18 times.

Why selected: KPType 2 diabetes: atypical diabetes in Africa

Document Type: Article
Source: Scopus

----

23. Mensah, G.A.
Epidemiology of stroke and high blood pressure in Africa
(2008) Heart, 94 (6), pp. 697-705. Cited 41 times.

Document Type: Review
Source: Scopus

----

24. Richard-Kadio, M., Yeo, S., Kossoko, H., Allah, C.-K., Assi-Dje Bi Dje, V.
Dupuytren's contracture. A report of three cases in Black Africans [La maladie de Dupuytren. À propos de trois cas chez le Noir Africain]
(2008) Chirurgie de la Main, 27 (1), pp. 40-42.

Document Type: Article
Source: Scopus

----

25. Carmoi, T., Verret, C., Debonne, J.M., Klotz, E.
Management of type 2 diabetes in subsaharan Africa: update and perspective [Prise en charge du diabète de type 2 en Afrique subsaharienne: constats actuels et perspectives.]
(2007) Médecine tropicale : revue du Corps de santé colonial, 67 (6), pp. 601-606.

Document Type: Article
Source: Scopus

----

26. Gombet, T., Steichen, O., Plouin, P.-F.
Hypertensive disease in subjects born in sub-Saharan Africa or in Europe referred to a hypertension unit: A cross-sectional study [Maladie hypertensive des personnes nées en Afrique subsaharienne adressées à un service spécialisé: Étude transversale comparative]
(2007) Bulletin de l'Academie Nationale de Medecine, 191 (8), pp. 1745-1755. Cited 3 times.

Document Type: Article
Source: Scopus

27.Sobngwi, E., Effoe, V., Boudou, P., Njamen, D., Gautier, J.-F., Mbanya, J.-C.
Waist circumference does not predict circulating adiponectin levels in sub-Saharan women
(2007) Cardiovascular Diabetology, 6, art. no. 31, . Cited 13 times.

Document Type: Article
Source: Scopus

----

28. Elvin-Lewis, M.
Evolving concepts related to achieving benefit sharing for custodians of traditional knowledge
(2007) African Journal of Traditional, Complementary and Alternative Medicines, 4 (4), pp. 443-468. Cited 1 time.

Document Type: Review
Source: Scopus

----

29. Seedat, Y.K.
Impact of poverty on hypertension and cardiovascular disease in sub-Saharan Africa
(2007) Cardiovascular Journal of Africa, 18 (5), pp. 316-320. Cited 10 times.

Document Type: Review
Source: Scopus

----

30. Kalk, W.J., Joffe, B.I.
Differences in coronary heart disease prevalence and risk factors in African and White patients with type 2 diabetes
(2007) Diabetes Research and Clinical Practice, 77 (1), pp. 107-112. Cited 9 times.

Document Type: Article
Source: Scopus

----

31. Beran, Mr, Yudkin, J.S.
Diabetes care in sub-Saharan Africa
(2006) Lancet, 368 (9548), pp. 1689-1695. Cited 68 times.

Document Type: Review
Source: Scopus

----

32. Kengne, A.P., Anderson, C.S.
The neglected burden of stroke in Sub-Saharan Africa
(2006) International Journal of Stroke, 1 (4), pp. 180-190. Cited 30 times.

Document Type: Review
Source: Scopus

----

40. Steyn, K., Sliwa, K., Hawken, S., Commerford, P., Onen, C., Damascene, A., Ounpuu, S., Yusuf, S.
Risk factors associated with myocardial infarction in Africa: The INTERHEART Africa Study
(2005) Circulation, 112 (23), pp. 3554-3561. Cited 139 times.

Document Type: Article
Source: Scopus

----

41. Otieno, C.F., Kayima, J.K., Omonge, E.O., Oyoo, G.O.
Diabetic ketoacidosis: Risk factors, mechanisms and management strategies in sub-Saharan Africa: A review
(2005) East African Medical Journal, 82 (12 SUPPL.), pp. S197-S203. Cited 14 times.

Comment: to be considered for hand search

Document Type: Article
Source: Scopus

----

42. Kengne, A.P., Amoah, A.G.B., Mbanya, J.-C.
Cardiovascular complications of diabetes mellitus in sub-Saharan Africa
(2005) Circulation, 112 (23), pp. 3592-3601. Cited 66 times.

Document Type: Review
Source: Scopus

----

43. Lemogoum, D., Degaute, J.-P., Bovet, P.
Stroke prevention, treatment, and rehabilitation in Sub-Saharan Africa
(2005) American Journal of Preventive Medicine, 29 (5 SUPPL. 1), pp. 95-101. Cited 32 times.

Document Type: Review
Source: Scopus

----

44. Young, B.A., Katon, W.J., Von Korff, M., Simon, G.E., Lin, E.H.B., Ciechanowski, P.S., Bush, T., Oliver, M., Ludman, E.J., Boyko, E.J.
Racial and ethnic differences in microalbuminuria prevalence in a diabetes population: The pathways study
(2005) Journal of the American Society of Nephrology, 16 (1), pp. 219-228. Cited 42 times.

Document Type: Article
Source: Scopus

----

45. McGlynn, K.A., London, W.T.
Epidemiology and natural history of hepatocellular carcinoma
(2005) Best Practice and Research: Clinical Gastroenterology, 19 (1 SPEC. ISS.), pp. 3-23. Cited 155 times.

Document Type: Review
Source: Scopus

----

46. Mauvais-Jarvis, F., Sobngwi, E., Porcher, R., Riveline, J.-P., Kevorkian, J.-P., Vaisse, C., Charpentier, G., Guillausseau, P.-J., Vexiau, P., Gautier, J.-F.
Ketosis-Prone Type 2 Diabetes in Patients of Sub-Saharan African Origin: Clinical Pathophysiology and Natural History of β-Cell Dysfunction and Insulin Resistance

(2004) Diabetes, 53 (3), pp. 645-653. Cited 91 times.

Document Type: Article
Source: Scopus

----

48. Walker, A.R.P., Walker, B.F., Segal, I.
Some puzzling situations in the onset, occurrence and future of coronary heart disease in developed and developing populations, particularly such in sub-Saharan Africa
(2004) Journal of The Royal Society for the Promotion of Health, 124 (1), pp. 40-46. Cited 13 times.

Document Type: Review
Source: Scopus

----

49. Sobngwi, E., Lubin, V., Ury, P., Timsit, F.-J., Gautier, J.-F., Vexiau, P.
Adrenal insufficiency and diabetes mellitus secondary to the use of topical corticosteroids for cosmetic purpose
(2003) Annales d'Endocrinologie, 64 (3), pp. 202-204. Cited 11 times.

Document Type: Article
Source: Scopus

----

50. Osei, K., Schuster, D.P., Amoah, A.G.B., Owusu, S.K.
Pathogenesis of type 1 and type 2 diabetes mellitus in sub-saharan africa: Implications for transitional populations
(2003) Journal of Cardiovascular Risk, 10 (2), pp. 85-96. Cited 22 times.

Document Type: Review
Source: Scopus

----

51. Motala, A.A., Omar, M.A.K., Pirie, F.J.
Epidemiology of type 1 and type 2 diabetes in africa
(2003) Journal of Cardiovascular Risk, 10 (2), pp. 77-83. Cited 45 times.

Document Type: Review

Source: Scopus

----

52. Naicker, S.
End-stage renal disease in sub-Saharan and South Africa
(2003) Kidney International, Supplement, 63 (83), pp. S119-S122. Cited 69 times.

Document Type: Conference Paper
Source: Scopus

----

60. Sobngwi, E., Vexiau, P., Levy, V., Lepage, V., Mauvais-Jarvis, F., Leblanc, H., Mbanya, J.C., Gautier, J.F.
Metabolic and immunogenetic prediction of long-term insulin remission in African patients with atypical diabetes
(2002) Diabetic Medicine, 19 (10), pp. 832-835. Cited 35 times.

Document Type: Article
Source: Scopus

----

61. Wanjohi, F.W., Otieno, F.C.F., Ogola, E.N., Amayo, E.O.
Nephropathy in patients with recently diagnosed type 2 diabetes mellitus black Africans
(2002) East African Medical Journal, 79 (8), pp. 399-404. Cited 7 times.

Document Type: Article
Source: Scopus

62.Kasvosve, I., Gangaidzo, I.T., Gomo, Z.A.R., Gordeuk, V.R.
African iron overload
(2000) Acta Clinica Belgica, 55 (2), pp. 88-93. Cited 11 times.

Document Type: Article
Source: Scopus

----

63. Essén, B., Hanson, B.S., Östergren, P.-O., Lindquist, P.G., Gudmundsson, S.
Increased perinatal mortality among sub-Saharan immigrants in a city-population in Sweden

(2000) Acta Obstetricia et Gynecologica Scandinavica, 79 (9), pp. 737-743. Cited 87 times.

Document Type: Article
Source: Scopus

----

64. Swai, A.B.M., Lutale, J.L., McLarty, D.G.
Prospective study of incidence of juvenile diabetes mellitus over 10 years in Dar es Salaam, Tanzania
(1993) British Medical Journal, 306 (6892), pp. 1570-1572. Cited 27 times.

Document Type: Article
Source: Scopus

----

65. Konje, J.C., Odukoya, O.A., Otolorin, E.O., Ewings, P.D., Ladipo, O.A.
Carbohydrate metabolism before and after norplantR removal
(1992) Contraception, 46 (1), pp. 61-69. Cited 12 times.

Document Type: Article
Source: Scopus

----

66. Harries, A.
Some clinical aspects of HIV infection in Africa.
(1992) Africa health, 14 (5), pp. 10-11.

Document Type: Article
Source: Scopus

----

67. Kamau, R.K., Maina, F.W., Kigondu, C., Mati, J.K.
The effect of low-oestrogen combined pill, progestogen-only pill and medroxyprogesterone acetate on oral glucose tolerance test.
(1990) East African Medical Journal, 67 (8), pp. 550-555. Cited 3 times.

Document Type: Article
Source: Scopus

----

68. Walker, A.R., Isaacson, C., Segal, I.
Third World policies and realities.
(1981) Lancet, 1 (8223), p. 779.

Document Type: Letter
Source: Scopus

==
